# Exploring the national prevalence of mental health risk, multimorbidity and the associations thereof: a repeated cross-sectional panel study

**DOI:** 10.3389/fpubh.2023.1217699

**Published:** 2023-10-18

**Authors:** Ashleigh Craig, Witness Mapanga, Asanda Mtintsilana, Siphiwe Dlamini, Shane Norris

**Affiliations:** ^1^SAMRC/Wits Developmental Pathways for Health Research Unit, Faculty of Health Sciences, University of the Witwatersrand, Johannesburg, South Africa; ^2^Strengthening Oncology Services Research Unit, Faculty of Health Sciences, University of the Witwatersrand, Johannesburg, South Africa; ^3^DSI-NRF Centre of Excellence in Human Development, University of Witwatersrand, Johannesburg, South Africa; ^4^School of Physiology, Faculty of Health Sciences, University of the Witwatersrand, Johannesburg, South Africa; ^5^School of Human Development and Health, University of Southampton, Southampton, United Kingdom

**Keywords:** depression, anxiety, ACE, mental health, multimorbidity, South Africa, national representative survey

## Abstract

**Objective and methods:**

South Africans were affected by the COVID-19 pandemic and resultant economic hardships. As a result, mental health within this region may have worsened. Therefore, using large scale nationally representative data, we repeated the cross-sectional panel study to investigate mental health risk post COVID-19 to explore mental health and multimorbidity and to examine the relationship between adverse childhood experiences (ACEs) and comorbid health conditions in South African adults (aged 18 years and older).

**Results:**

Post-pandemic, 26.2, 17.0, and 14.8% of the South African respondents reported being probably depressed, anxious and had suffered high exposure to early life adversity, respectively. Nationally, the prevalence of mental health across the country remained alarmingly high when compared to Panel 1. The prevalence of multimorbidity (2 or more chronic morbidities) among the South African population was reported at 13.9%, and those with 2 or more morbidities were found to have increased odds of early adversity, irrespective of differing socio-demographics. Furthermore, early adversity was also associated with multimorbidity partly via mental health.

**Conclusion:**

This repeated cross-sectional national study reiterated that the prevalence of mental health across South African adults aged 18 years and older is widespread. Mental health remains worryingly high post-pandemic where more than a quarter of respondents are probably depressed, nearly one in every five respondents are anxious, and 14.8% reported high exposure ACEs. Public health interventions need to be upscaled with efforts to reduce the incidence of early adversity that may have the ability to lower adverse health outcomes and mental ill-health in adulthood.

## Introduction

Beyond the human tragedy, the COVID-19 pandemic has decimated economies, ravaged livelihoods, and triggered what is now widely recognised as the most serious global economic crisis since World War II (1). With most high-income countries (HICs) straining to cope under the weight of the COVID-19 aftermath, low-to-middle income countries (LMICs) are further overextended due to their deep-rooted low-resource settings (2). Mental health associates with morbidity and mortality in adulthood (3) and is related to poverty, inequality, and other social and economic stressors (4). Consequently, post COVID-19, mental health risks in LMICs may have worsened due to the economic challenges and rising costs of living.

A 2021 cross-sectional national panel survey (post COVID-19 wave 3) in South Africa (SA) showed an alarming prevalence of probable depression, anxiety, and high early childhood adversity that varied significantly across its’ nine provinces (5). Furthermore, the panel study found that depression and anxiety were more frequently reported among adults who were: retired and older than 65 years; widowed, divorced, or separated; living in a metropolitan area, and/or with only a basic level of education (5). These characteristics are more often linked to economic hardship and poverty (6). The survey also showed that probable depression and/or anxiety in South African adults was greater in those who reported high exposure to adverse childhood experiences (ACEs), independent of socio-economic status (SES) and demographics (5).

South Africa was considered the regional epicentre of COVID-19 across sub-Saharan Africa (7) and faced the pandemic with an already weakened economy (8). In efforts to prevent an unmanageable surge, SA, as in many countries, implemented stringent public and social restrictions. Many believe that these control measures (i.e., lockdown; curfew; prohibited sale of alcohol and tobacco products; and closure of non-essential retailers) may have caused more economic loss and difficulties (7). Studies exploring mental health prior to, during and post outbreak reported that mental health risk was highest during the outbreak period (9–11), while some show an escalation post outbreak due to the ongoing global economic turmoil (12). Therefore, we repeated the cross-sectional panel study 9 months later following Panel 1 and relaxation of COVID-19 restrictions to investigate mental health risk during exacerbated economic pressure, to explore mental health and multimorbidity, and lastly, to examine the relationship between early adversity and comorbid health conditions in South African adults (aged 18 years and older) using large-scale, nationally representative data.

## Methodology

### Study design

**Figure 1:**
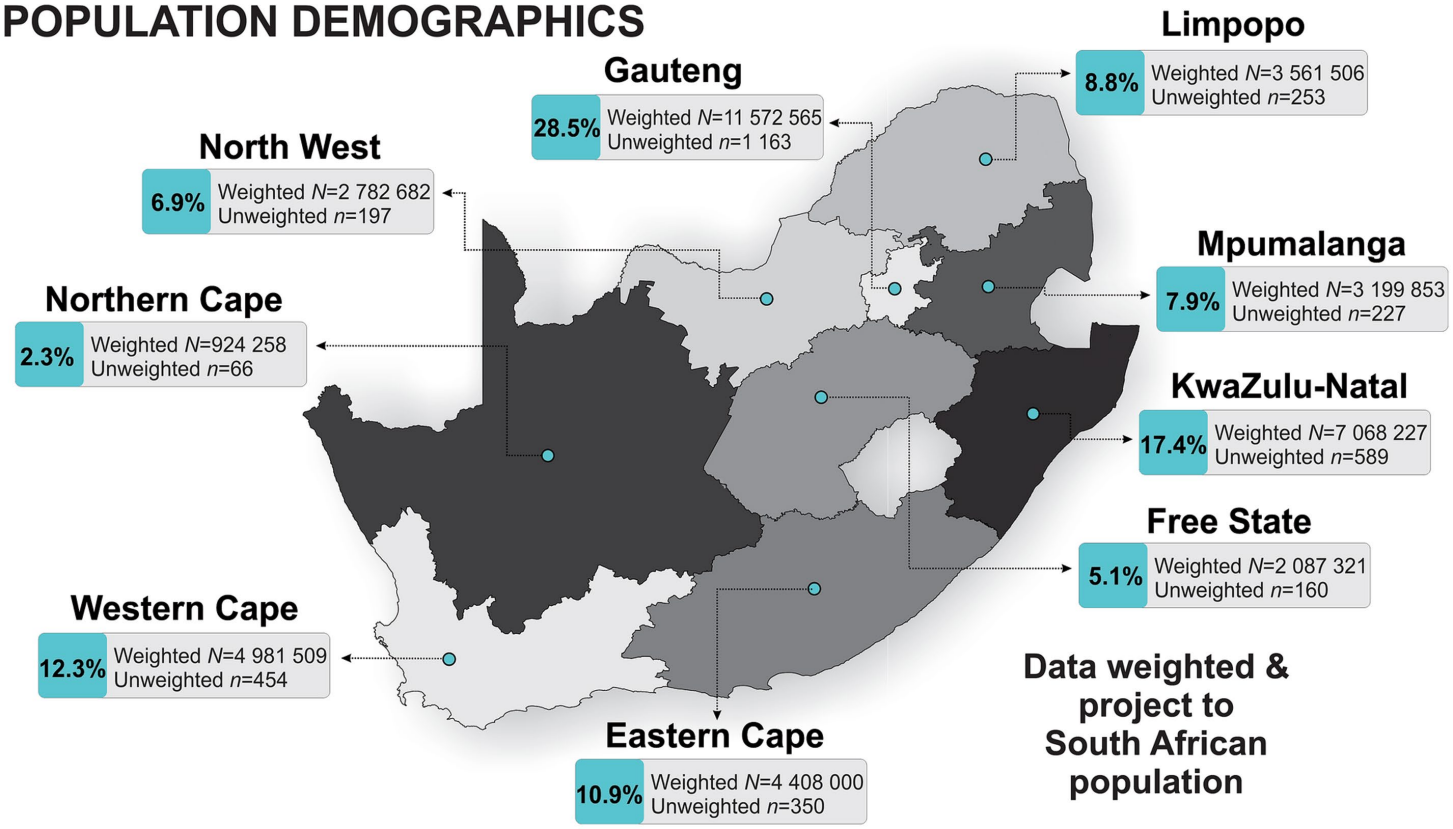
Population demographics outlining the nine provinces of South Africa.

The current study is a repeated cross-sectional panel (May–June 2022; now referred to as Panel 2), that followed the first nationally representative panel surveyed in September–October 2021 (now referred to as Panel 1: *n* = 3,402) (5). In Panel 2, interviews were conducted with 3,459 respondents across the nine provinces of SA (Figure 1). During the data collection of Panel 2, COVID-19 lockdown restrictions had eased across the country compared to that of Panel 1 (Supplementary Table S1).

The study obtained approval from the Human Research Ethics Committee (Non-Medical) of the University of the Witwatersrand, SA (H21/06/36). Written informed consent was obtained from each respondent.

### Survey

Data collection followed the same procedures as outlined in Panel 1 (5). In brief, data collection took place through face-to-face interviews to administer a survey with the use of computer-assisted personal interviewing technology. A team of experienced fieldworkers moved from household to household across the nine provinces in the country until the target number was reached. For a particular community, enrolment was thus executed via a targeted approach to ensure generalisability. The survey included questions related to respondent and household demographics. Province and community size (metropolitan, city/town and rural/village) were recorded and information collection on household assets, age, gender, employment status, marital status, education attained, and health-related questions (mental health and multimorbidity). A household asset score was computed in alignment with the Demographic and Health Surveys household questionnaire and used as an indicator of SES in this study. This included a tally of all major operational household amenities (e.g., refrigerator, washing machine, television, computer etc.). In this repeated cross-sectional study, household asset score tertiles were computed and used as an indicator of economic differentiation (13–15).

To assess depression, the Patient Health Questionnaire (PHQ-9) scale was used (16). This scale consists of nine questions with responses recorded on a four-point Likert scale ranging from 0 (“Not at all”) to 3 (“Nearly every day”). The level of depression was then categorised into five groups, which were, minimal, mild, moderate, moderately severe, and severe based on scoring in the range of 0–4, 5–9, 10–14, 15–19, and 20–27, respectively (16). Binary classification of probable depression was defined by a PHQ-9 score of 10 or greater.

Similarly, to assess anxiety, the Generalised Anxiety Disorder (GAD-7) scale was used (16). The GAD-7 scale consists of seven questions with a four-point Likert response scale ranging from 0 (“Not at all”) to 3 (“Nearly every day”). The level of anxiety was categorised into four groups, which were minimal, mild, moderate, and severe based on scoring in the range of 0–4, 5–9, 10–14, and 15–21, respectively (16). Binary classification of probable anxiety was defined by a GAD-7 score of 10 or greater (17).

Adverse childhood experiences were measured through a 12-item ACE questionnaire, which is an individual’s retrospective report of specific adversities experienced over the first 18 years of their life (18). Exposure to ACEs was operationalised using 12 types of experiences falling within three categories: emotional and/or physical abuse, sexual abuse, or household dysfunction (18). An overall ACE score was calculated based on the number of affirmative responses. The ACE score was categorised into three exposure groups based on scoring 0 (no exposure); 1–3 (intermediate exposure) and 4–12 (high exposure), respectively (19).

To assess the overall health of the respondent, respondents were asked a series of health-related questions about known chronic conditions [i.e., heart attack; stroke, high cholesterol, diabetes, overweight/obesity, HIV/AIDS, asthma/chronic obstructive pulmonary disease, sore joints/muscle problems (i.e., arthritis, gout), tuberculosis, cancer, liver disease, mental health (i.e., depression, anxiety and bipolar), chronic kidney disease and hypertension/high blood pressure]. An overall multimorbidity score was calculated based on the sum of chronic conditions including mental health. Multimorbidity is frequently interpreted as the coexistence of several confirmed health conditions, often chronic, an individual experiences. The multimorbidity score was therefore categorised into three groups based on those respondents who reported null or one ailment (group 1: 0–1 morbidity); those who present with comorbidity (i.e., more than 1 aliment; group 2: 2 morbidities); and those who present with multimorbidity (i.e., more than two ailments; group 3: ≥3 morbidities), respectively.

### Statistical analyses

For all statistical analyses, IBM® SPSS® version 28 (IBM Corporation, Armonk, New York) and GraphPad Prism version 5.03 for Microsoft® Windows (GraphPad Software, San Diego, California, United States) were used to analyse and plot the data. Additionally, QGIS (Penn Libraries, Philadelphia, PA, United States) was used to plot and scale the geographical location of the South African provinces.

All statistics were weighted to represent the most recent census of the South African population (18 years or older). The weighted matrix factored in age, sex, population group, home language and provincial distribution. Proportions across socio-demographics (age, sex, marital status, education level, employment, household assets and urbanicity) and provinces were determined with crosstabs with significant differences indicated by Chi-square tests and presented as percentages. Univariate and multivariable adjusted binary logistic regressions were performed to determine the odds of probable depression (PHQ-9 score ≥ 10), probable anxiety (GAD-7 score ≥ 10) and multimorbidity (chronic conditions excl. mental health) in adulthood with either ACEs or ACEs and socio-demographic contributors (age, sex, marital status, education level, employment, SES and urbanicity) as confounders.

Additionally, the past few decades have seen increasing acknowledgment of mental health risk as predictors for overall health in adulthood. Among these, depression and anxiety continue to be the most researched factor in the field. *A posteriori* simple mediation analysis was therefore used to determine whether probable depression and/or probable anxiety mediate the relationship between multimorbidity and ACE score. Models were adjusted for age, sex, education attained, employment status, marital status, SES and urbanicity. In each model, a dependent variable (multimorbidity), independent variable (ACE score) and mediator variable (depression and/or probable anxiety) were included to calculate the unstandardised regression coefficients and to generate total, direct and indirect effects. Full mediation was classified when there were significant total and indirect effects and non-significant direct effects. When the total, indirect and direct effects were significant, a partial mediation was considered to have occurred. Inconsistent mediation was present when only an indirect effect was significant. Bootstrapping of 5,000 replications was used to generate normal-based bootstrapped confidence intervals around the indirect effect. Analysis was completed using the PROCESS macro (version 4) for IBM SPSS Statistics (version 28, Statistical Package for the Social Sciences, Chicago, IL, United States) (20). In all statistical analyses, probable depression, probable anxiety and multimorbidity (continuous or binary) were considered dependent outcome variables, while ACE score and/or socio-demographic determinants were considered independent variables in the various models.

To avoid multicollinearity in our statistical models, mental health was excluded from the multimorbidity score where correlations with mental health aspects were involved (i.e., any statistical models that included depression, anxiety, or ACEs as either a dependent or independent variable).

## Results

### Repeated cross-sectional analysis

The full characteristics and associations of Panel 1 have been described elsewhere (5). In this current sample, a total of 3,459 respondents (female: 50.7%; male: 49.3%) were included for repeated cross-sectional analysis (Table 1). Respondents were predominantly young adults aged 25–34 years (29.4%). The largest proportion of respondents were those who reported a marital status of single (57.8%), employed (51.6%), an education level of graduated high school or equivalent (54.2%), and resided in the Gauteng province [*n* = 1,163 (28.5%); Figure 1]. When compared to Panel 1 (2021), the only difference in respondent demographics, was respondents that participated in this survey (Panel 2) were predominantly younger (25-34 vs. <44 years).

**Table 1:** General descriptive of the South African survey respondents (*n* = 3,459).

		Unweighted	Weighted			Unweighted	Weighted			Unweighted	Weighted
Age categories				Depression categories				Smoke			
18–24 years	%	16.5	20.2	Minimal	%	49.3	49.2	Yes	%	95.6	96.1
25–34 years	%	29.4	28.7	Mild	%	24.5	24.6	No	%	4.4	3.9
35–44 years	%	27.3	22.1	Moderate	%	16.5	16.7				
45–54 years	%	17.1	15.0	Moderately severe	%	7.3	7.1	Alcohol			
55–64 years	%	7.1	10.5	Severe	%	2.5	2.4	Yes	%	36.6	65.8
65+ years	%	2.6	3.5	Probable depression	%	26.3	26.2	No	%	63.4	34.2
Education				Anxiety categories				Multimorbidity (chronic conditions incl. Mental health)			
Uneducated/Partial primary	%	1.8	2.9	Minimal	%	56.2	55.7	0–1 morbidity	%	87.3	86.1
Primary school	%	1.8	2.2	Mild	%	27.0	27.3	2 morbidities	%	6.7	7.0
Partial secondary	%	21.4	22.3	Moderate	%	13.1	13.3	3+ morbidities	%	6.1	6.9
NSC/Short course	%	54.2	52.8	Severe	%	3.7	3.7	Sex			
Tertiary	%	20.9	19.8	Probable anxiety	%	16.8	17.0	Male	%	49.3	47.8
Socio-economic status				ACE score				Female	%	50.7	52.2
Lower tertile	%	34.4	37.8	No exposure (*n* = 2095)	%	60.6	60.4				
Middle tertile	%	40.6	38.7	Intermediate exposure (*n* = 839)	%	24.3	24.8				
Upper tertile	%	25.0	23.4	High exposure (*n* = 524)	%	15.2	14.8				
Employment status				ACE types							
Unemployed	%	37.1	37.2	Household dysfunction	%	33.1	33.3				
Employed	%	51.6	48.9	Emotion/physical abuse	%	28.9	28.8				
Student	%	6.9	8.1	Sexual abuse	%	4.9	5.2				
Retired	%	4.4	5.8								
Marital status				Urbanicity							
Single	%	57.8	58.2	Metropolitan	%	57.8	49.3				
Married/Co-habit	%	36.0	34.9	City/Town	%	16.4	20.0				
Widowed/Divorced	%	6.2	6.9	Rural/Village	%	25.7	30.7				

Probable depression was categorised into five groups based on scoring in the range 0–4 (minimal), 5–6 (mild), 10–14 (moderate), 15–19 (moderately severe), and 20–27 (severe). Probable anxiety was categorised into four groups based on scoring in the range 0–4 (minimal), 5–9 (mild), 10–14 (moderate), and 15–21 (severe). The ACE score was categorised into three exposure groups based on scoring 0 (no exposure); 1–3 (intermediate exposure), and 4–12 (high exposure). Multimorbidity score was categorised into three groups based on those respondents who reported null or one ailment (0–1 morbidity); those with comorbidity (2 morbidities); and those with multimorbidity (3 morbidities). *n*, Number of participants; %, Percentage; ACE, Adverse childhood experience; NSC, National senior certificate.

#### Prevalence of mental health

**Figure 2:**
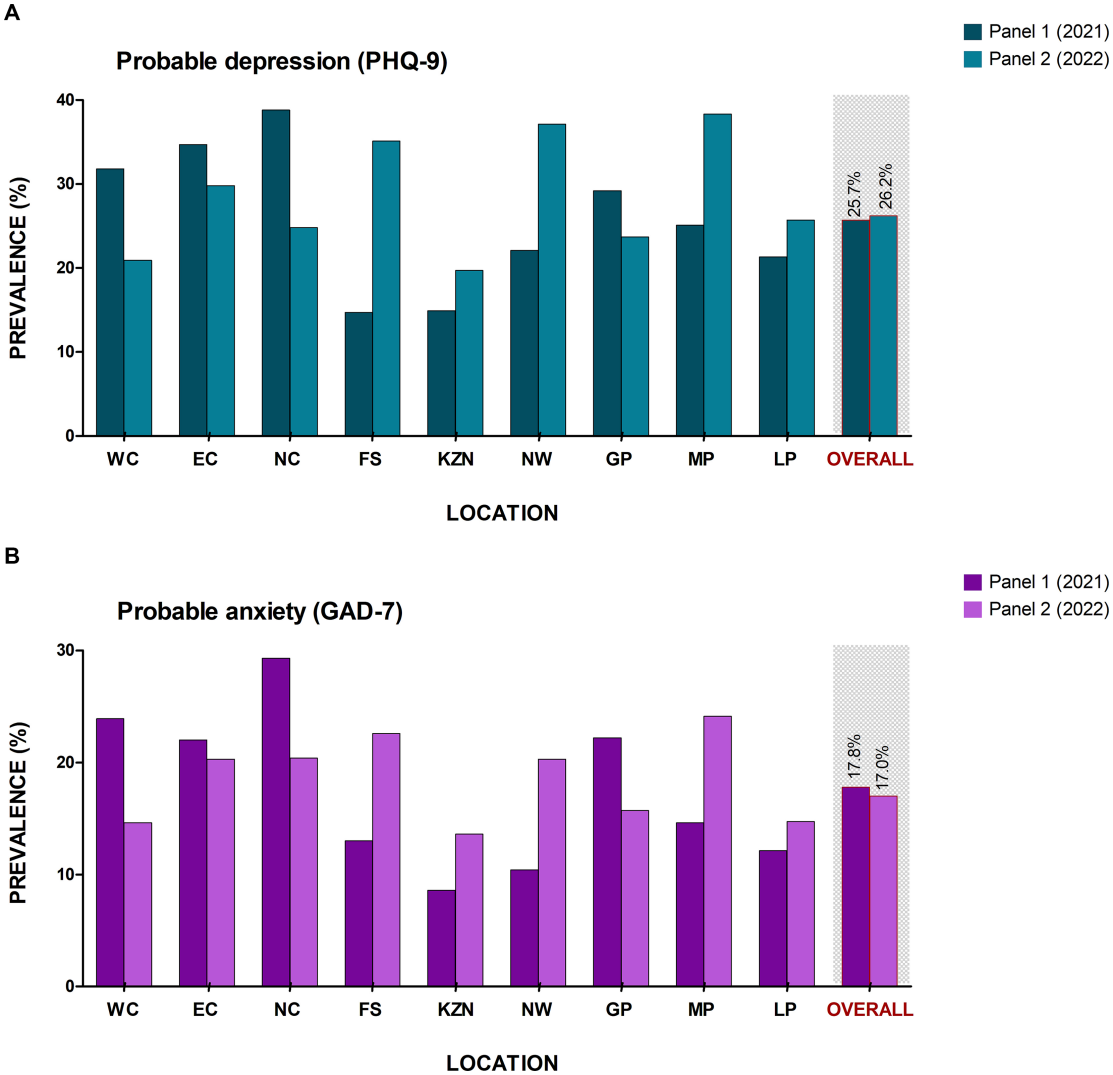
Prevalence of mental health risk across the South African provinces. **(A)** Probable depression and **(B)** Probable anxiety. NC, Northern Cape; WC, Western Cape; NW, North West; GP, Gauteng province; LP, Limpopo province; MP, Mpumalanga province; FS, Free state; KZN, Kwa-Zulu Natal; and EC, Eastern Cape.

Overall, the majority of respondents had a minimal risk PHQ-9 score (49.2%), while those scoring as mild, moderate, moderately severe, and severe were 24.6, 16.7, 7.1 and 2.4% respectively, which resulted in a prevalence of 26.2% of probable depression in SA (moderate, moderately severe and severe; Table 1; Figure 2). Contrary to Panel 1, Mpumalanga reported the highest prevalence of both probable depression (38.3%) and probable anxiety (22.6%), while Kwa-Zulu Natal reported the lowest prevalence of both probable depression (19.7%) and probable anxiety (13.6%) out of all nine provinces (Figure 2; Supplementary Table S2). Adverse childhood experience scores were recorded in the range of 0.752–2.76, with the highest mean ACE score (2.76) reported in the Northern Cape Province (SD: 2.48). Additionally, 1,146, 999 and 168 respondents reported specific ACE types such as household dysfunction, emotional/physical abuse and sexual abuse, respectively.

#### Socio-demographic correlates

Table 2 presents the prevalence of probable depression, probable anxiety and high ACE exposure among the study respondents stratified by socio-demographic determinants. In line with Panel 1 (2021), the prevalence of probable depression and anxiety was higher in women (≥17.4%); widowed, divorced, or separated (≥23.2%) and/or with only a basic level of education, i.e., completed primary school (≥24.8%). Respondents in Panel 2 also showed a comparable higher prevalence of probable depression and high ACE exposure in those respondents with an SES score in the lowest tertile (≥7.8%), while probable anxiety was higher in those respondents in the middle SES tertile (18.0%). In contrast to panel 1 though, probable depression was higher in those 34–44 years of age (28.4%) and those who were unemployed (28.8%). Respondents that reported the highest prevalence of probable anxiety were those who are retired (≥21.2%) and/or 65 years or older (≥23.3%). Of note, those in the highest exposure groups with an ACE score of 4 or greater were respondents who were single (15.6%) in the age range of 18–24 years (18.4%), currently unemployed (17.0%) and/or with a level of education of partially completing secondary school (19.0%).

**Table 2:** Socio-demographics of the South African adult population stratified by mental and overall health risk.

		Generalised anxiety disorder (GAD7)					Patient health questionnaire (PHQ9)						ACEs			Multimorbidity (chronic conditions incl. mental health)		
		Probable anxiety	Min	Mild	Moderate	Severe	Probable depression	Min	Mild	Moderate	Moderately severe	Severe	No exposure	Intermediate exposure	High exposure	0–1	2	3+
Age																		
18–24 years	%	17.8	56.1	26.1	13.8	4.0	24.3	46.9	28.8	15.5	6.6	2.2	53.8	27.8	18.4	93.0	3.5	3.5
25–34 years	%	13.9	57.5	28.6	10.8	3.1	23.4	50.9	25.7	15.2	6.6	1.6	61.4	25.6	13.0	91.9	4.0	4.1
35–44 years	%	20.0	53.1	26.8	15.2	4.8	30.0	47.2	22.8	18.2	8.4	3.4	60.7	22.7	16.6	90.5	4.8	4.8
45–54 years	%	16.0	58.5	25.5	12.3	3.7	26.9	51.3	21.9	17.0	7.5	2.4	62.8	24.6	12.6	80.6	11.6	7.8
55–64 years	%	17.3	52.7	30.1	14.8	2.5	28.4	50.0	21.6	19.5	6.6	2.3	61.6	23.5	14.9	68.9	13.6	17.5
65+ years	%	23.3	50.7	25.9	19.8	3.5	26.3	50.4	23.3	17.1	5.2	4.0	74.2	19.7	6.1	47.0	25.8	27.1
Sex																		
Male	%	16.4	57.7	25.9	12.5	3.9	25.6	50.4	24.0	15.9	7.3	2.4	61.1	24.4	14.5	88.2	5.2	6.6
Female	%	17.4	53.7	28.7	14.1	3.5	26.7	48.1	25.2	17.4	6.9	2.4	59.7	25.2	15.1	84.2	8.6	7.2
Marital status																		
Single	%	18.1	53.1	28.8	14.0	4.1	27.7	45.4	26.8	17.2	7.9	2.6	58.9	25.5	15.6	90.2	5.1	4.6
Married/Co-habit	%	14.0	61.2	24.7	11.5	2.5	22.3	56.5	21.2	15.5	5.1	1.7	62.2	23.8	14.0	83.1	8.1	8.9
Widowed/Divorced/Separated	%	23.2	48.7	28.1	16.8	6.4	32.5	44.3	23.2	18.4	10.0	4.1	64.4	24.3	11.3	67.3	16.7	16.0
Employment																		
Unemployed	%	18.7	51.8	29.5	14.5	4.2	28.8	43.2	28.0	17.6	8.0	3.2	56.6	26.0	17.5	89.3	5.1	5.6
Employed	%	15.4	58.3	26.3	12.1	3.3	24.2	53.6	22.3	16.0	6.5	1.7	62.0	24.6	13.4	85.8	7.2	6.9
Student	%	12.2	59.7	25.9	12.0	2.4	24.8	49.7	25.5	18.3	5.3	1.2	64.0	21.5	14.5	95.3	2.2	2.6
Retired	%	23.5	52.9	23.6	18.3	5.2	28.6	50.6	20.8	15.2	8.3	5.1	66.4	23.8	9.8	55.7	23.1	21.2
Education																		
Uneducated/ Partial primary	%	20.3	58.4	21.3	18.9	1.4	33.7	53.6	12.7	22.6	7.6	3.5	57.7	30.5	11.8	72.3	13.4	14.2
Primary school	%	24.8	47.1	28.2	20.9	3.9	36.2	46.1	17.6	14.7	16.7	4.8	61.2	27.3	11.5	66.0	17.5	16.5
Partial secondary	%	18.2	53.5	28.3	13.5	4.7	25.9	46.1	27.9	16.2	6.8	2.9	52.5	28.5	19.0	85.0	7.3	7.7
NSC/Short course	%	16.5	56.9	26.7	13.0	3.5	25.5	49.9	24.7	16.1	7.1	2.3	61.1	23.9	15.0	88.0	5.9	6.1
Tertiary	%	16.0	55.3	28.8	12.5	3.5	26.2	50.7	23.1	18.2	6.2	1.8	67.7	21.9	10.4	86.7	7.3	6.0
Urbanicity																		
Metropolitan	%	16.1	59.3	24.6	12.6	3.5	24.0	53.3	22.7	14.8	6.4	2.8	63.0	22.7	14.4	86.6	6.9	6.5
City/Town	%	20.5	52.0	27.5	16.7	3.8	29.4	46.2	24.4	20.8	6.8	1.8	58.3	24.4	17.3	84.7	7.4	7.9
Rural/Village	%	16.2	52.2	31.6	12.3	3.9	27.6	44.6	27.7	17.0	8.4	2.2	57.6	28.6	13.8	86.4	6.8	6.9
Socio-economic status																		
Lower tertile	%	17.8	53.3	28.9	14.7	3.1	28.9	45.6	25.6	18.9	8.0	2.0	56.8	25.8	17.4	85.9	6.2	7.8
Middle tertile	%	18.0	53.2	28.8	13.5	4.5	27.3	46.7	26.1	18.0	6.6	2.7	59.7	26.1	14.2	87.0	6.7	6.5
Upper tertile	%	14.2	63.5	22.2	10.9	3.3	20.1	59.36	20.5	11.1	6.4	2.6	67.3	21.2	11.5	85.1	8.6	6.3
Lifestyle risk																		
Smoke (yes)	%	17.3	56.1	26.7	13.2	4.1	25.2	49.5	25.3	16.8	5.9	2.5	52.8	25.2	22.0	86.8	7.0	6.2
Alcohol (yes)	%	17.5	54.6	27.9	13.8	3.7	26.0	47.8	26.3	16.2	7.7	2.1	50.9	27.5	21.6	85.8	6.4	7.9

Probable depression was categorised into five groups based on scoring in the range 0–4 (minimal), 5–6 (mild), 10–14 (moderate), 15–19 (moderately severe), and 20–27 (severe). Probable anxiety was categorised into four groups based on scoring in the range 0–4 (minimal), 5–9 (mild), 10–14 (moderate), and 15–21 (severe). The ACE score was categorised into three exposure groups based on scoring 0 (no exposure); 1–3 (intermediate exposure) and 4–12 (high exposure). Multimorbidity score was categorised into three groups based on those respondents who reported null or one ailment (0–1 morbidity); those with comorbidity (2 morbidities); and those with multimorbidity (3 morbidities). %, Percentage; ACEs, Adverse childhood experiences; PHQ9, Patient health questionnaire; GAD7, Generalised anxiety disorder.

#### Associations of probable depression, probable anxiety, ACE score, multimorbidity and socio-demographics

**Figure 3:**
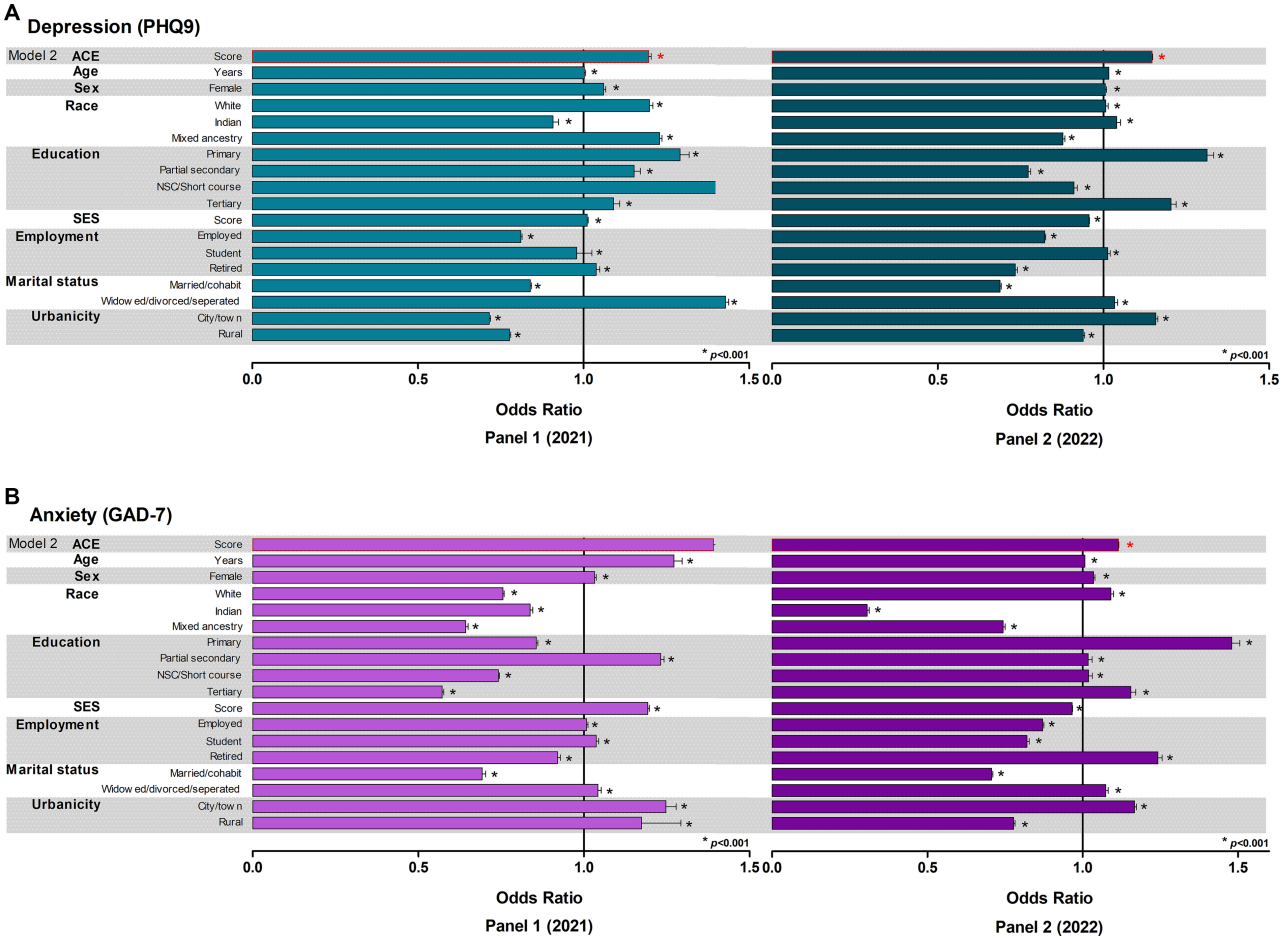
Binary logistic regressions to determine the odds of having **(A)** probable depression or **(B)** probable anxiety in adulthood. SES, socioeconomic status.

We repeated the univariate and multivariable adjusted binary logistic regressions (Figure 3; Supplementary Table S3) as seen in Panel 1 to corroborate the odds of having either probable depression or probable anxiety with higher levels of ACE exposure (model 1), or having ACE exposure, independent of socio-demographic characteristics (age, sex, marital status, education level, employment, SES and urbanicity; model 2). In model 1, we confirmed the results determined in Panel 1. The likelihood of having probable depression (OR, 1.15 [95% CI 1.148; 1.149]) or probable anxiety (OR, 1.12 [95% CI 1.116; 1.117]) increased by >12% with each standard deviation increase in the ACE score (*p* < 0.001). Similarly, in model 2, as seen in model 2 of Panel 1, the odds of either probable depression or probable anxiety increased with each standard deviation increase in the ACE score (all *p* < 0.001), independent of other socio-demographic determinants.

### Additional cross-sectional analysis

**Figure 4:**
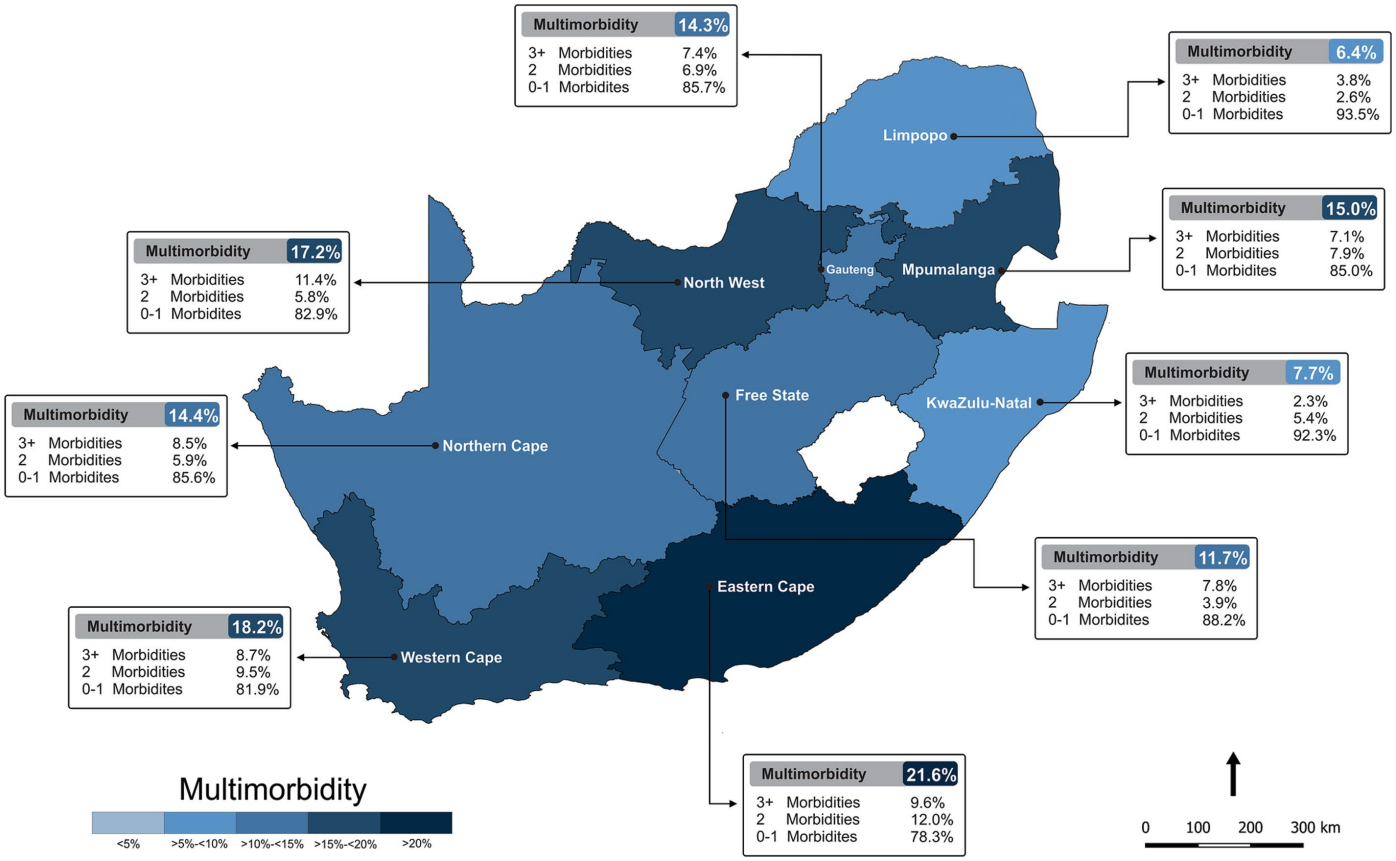
Prevalence of multimorbidity (chronic conditions incl. mental health) across South Africa.

The number of morbidities (chronic conditions incl. mental health) varied significantly across the country (Supplementary Table S2). The North West Province reported the highest proportion of respondents who described 3 or more morbidities (11.4%) with the Eastern Cape Province reporting the highest prevalence in multimorbidity (two or more morbidities) at 21.6% (Figure 4). Furthermore, multimorbidity of three morbidities or more was highest among women (7.2%), respondents with an SES score in the lowest tertile (7.8%), marital status as widowed, divorced or separated (16.0%) and employment status of retired (21.2%) and/or 65 years or older (27.1%), with only have completed primary school (16.5%). Introspectively, several chronic conditions significantly differed across the provinces (Supplementary Table S4). Respondents who resided in the North West province reported the highest prevalence in seven out of the 14 chronic conditions included in the multimorbidity score [stroke (5.3%), HIV/AIDS (10.7%), asthma/lung disease (10.8%), tuberculosis (5.6%), cancer (6.7%), liver disease (5.4%) and chronic kidney disease (4.5%)] when compared to respondents in the other eight provinces.

A series of regressions (univariate and multivariable adjusted binary logistic; Table 3) were carried out to determine if ACE exposure will positively predict multimorbidity (chronic conditions excl. mental health). We, therefore, determined the odds of having a higher number of morbidities with higher levels of ACE exposure (model 1), or having ACE exposure, independent of socio-demographic characteristics (model 2). In model 1, we determined that, respondents who reported having 0–1 morbidities were 80% less likely (OR, 0.804 [95% CI 0.804; 0.805]) to have experienced ACEs (*p* < 0.001). However, the likelihood of having two morbidities or three or more morbidities increased by 10% (OR, 1.10 [95% CI 1.092; 1.093]) or 32% (OR, 1.32 [95% CI 1.314; 1.315]), respectively, with each standard deviation increase in the ACE score (*p* < 0.001). In model 2, we found a similar pattern of increasing likelihood across the multimorbidity groups with respondents who reported having 0–1 morbidities were 76% less likely (OR, 0.759 [95% CI 0.759; 0.759]) to have experienced ACEs while the odds of having either two morbidities (OR, 1.13 [95% CI 1.129; 1.130]) or three or more morbidities (OR, 1.39 [95% CI 1.384; 1.386]) increased with each standard deviation increase in the ACE score, independent of other socio-demographic determinants (all *p* < 0.001).

**Table 3:** Logistic regressions to determine the odds of multimorbidity (chronic conditions excl. mental health) in adulthood.

			Multimorbidity (chronic conditions excl. mental health) 0–1 morbidities (*n* = 3,018)			Multimorbidity (chronic conditions excl. mental health) 2 morbidities (*n* = 221)			Multimorbidity (chronic conditions excl. mental health) 3+ morbidities (*n* = 201)		
			OR	(95% Cl)	*p* value	OR	(95% Cl)	*p* value	OR	(95% Cl)	*p* value
**Model 1**	ACE	Score	0.804	(0.804; 0.805)	**<0.001**	1.10	(1.092; 1.093)	**<0.001**	1.32	(1.314; 1.315)	**<0.001**
**Model 2**	ACE	Score	0.759	(0.759; 0.759)	**<0.001**	1.13	(1.129; 1.130)	**<0.001**	1.39	(1.384; 1.386)	**<0.001**
	Age	Years	0.943	(0.943; 0.943)	**<0.001**	1.05	(1.044; 1.045)	**<0.001**	1.06	(1.062; 1.062)	**<0.001**
	Sex	Male		(reference)		(reference)				(reference)	
		Female	0.680	(0.679; 0.681)	**<0.001**	1.76	(1.756; 1.765)	**<0.001**	1.09	(1.087; 1.093)	**<0.001**
	Education	Uneducated/Partial primary		(reference)		(reference)				(reference)	
		Primary	0.493	(0.490; 0.496)	**<0.001**	1.82	(1.810; 1.839)	**<0.001**	1.48	(1.466; 1.491)	**<0.001**
		Partial secondary	0.934	(0.929; 0.938)	**<0.001**	1.02	(1.016; 1.029)	**<0.001**	1.06	(1.061; 1.075)	**<0.001**
		NSC/Short course	0.825	(0.821; 0.829)	**<0.001**	1.12	(1.110; 1.124)	**<0.001**	1.18	(1.179; 1.194)	**<0.001**
		Tertiary	0.898	(0.893; 0.903)	**<0.001**	0.952	(0.945; 0.959)	**<0.001**	1.21	(1.205; 1.223)	**<0.001**
	SES	Score	0.982	(0.981; 0.982)	**<0.001**	1.04	(1.035; 1.036)	**<0.001**	0.997	(0.997; 0.998)	**0.003**
	Employment	Unemployed		(reference)		(reference)				(reference)	
		Employed	0.716	(0.714; 0.718)	**<0.001**	1.49	(1.488; 1.498)	**<0.001**	1.16	(1.157; 1.165)	**<0.001**
		Student	0.876	(0.870; 0.881)	**<0.001**	1.01	(0.997; 1.013)	**<0.001**	1.15	(1.139; 1.159)	**<0.001**
		Retired	0.509	(0.507; 0.511)	**<0.001**	2.00	(1.990; 2.010)	**<0.001**	1.38	(1.368; 1.382)	**<0.001**
	Marital status	Single		(reference)		(reference)				(reference)	
		Married/Co-habit	0.958	(0.956; 0.960)	**<0.001**	0.865	(0.862; 0.868)	**<0.001**	1.37	(1.363; 1.372)	**<0.001**
		Widowed/Divorced/Separated	0.734	(0.731; 0.736)	**<0.001**	1.09	(1.083; 1.093)	**<0.001**	1.64	(1.634; 1.650)	**<0.001**
	Urbanicity	Metropolitan		(reference)		(reference)				(reference)	
		City/Towns	0.884	(0.882; 0.887)	**<0.001**	1.03	(1.029; 1.036)	**<0.001**	1.14	(1.133; 1.142)	**<0.001**
		Rural/Village	0.840	(0.838; 0.843)	**<0.001**	1.28	(1.279; 1.289)	**<0.001**	1.05	(1.050; 1.058)	**<0.001**

Model 1: regression unadjusted. Model 2: regression adjusted for socio-demographics. n, Number of participants; SES, Socioeconomic status. Bold values denote statistical significance (*p* < 0.05). Multimorbidity score was categorised into three groups based on those respondents who reported null or one ailment (0–1 morbidity); those with comorbidity (2 morbidities); and those with multimorbidity (3 morbidities).

**Figure 5:**
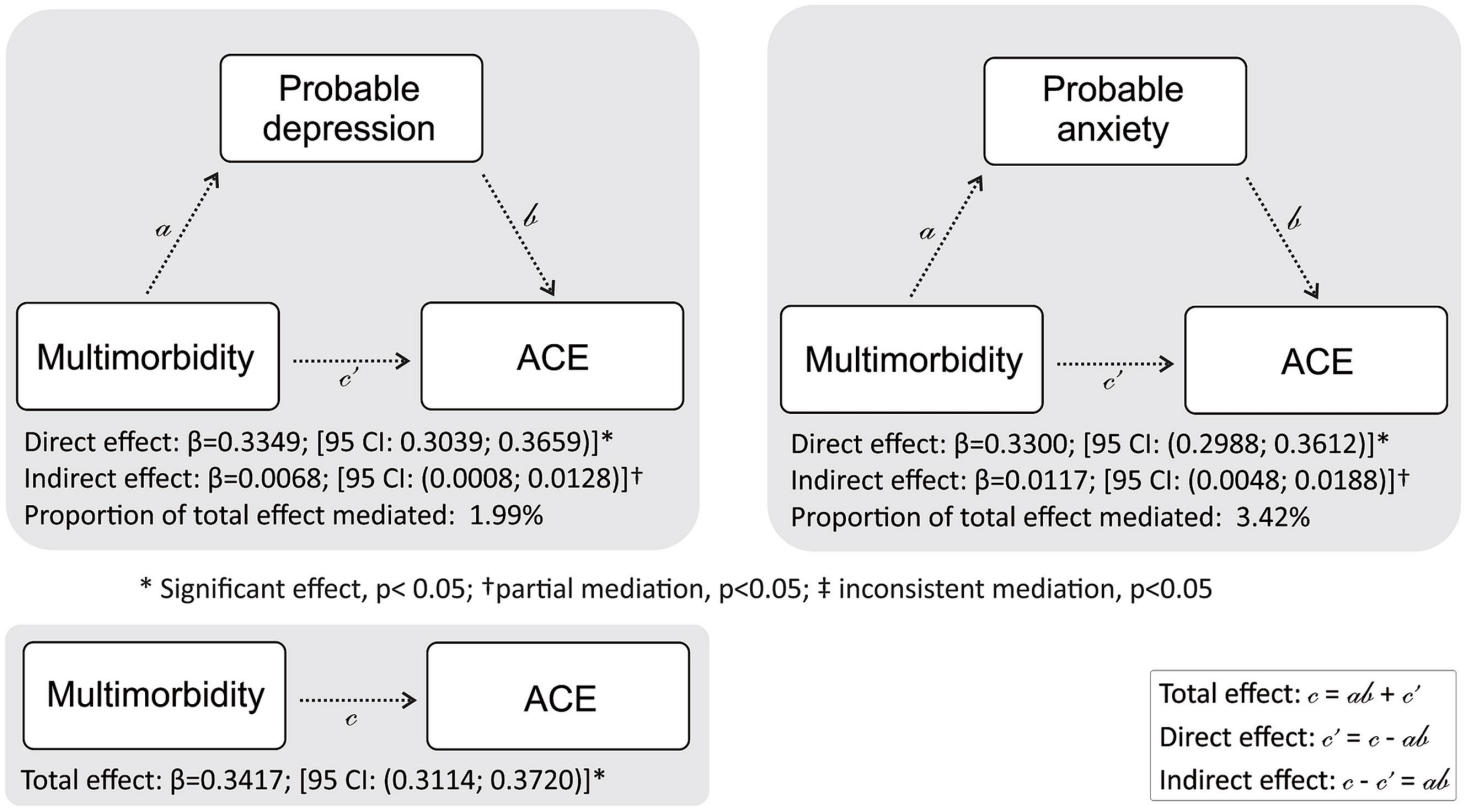
Mediation analysis in the study population to determine if probable depression or probable anxiety mediate the association between multimorbidity (chronic conditions excl. mental health) and ACE with adjustments made for *priori* covariates. Covariates in the model include age, sex, employment status, education attained, socio-economic status, marital status, and urbanicity.

Furthermore, a mediation analysis (Figure 5) was carried out to determine if probable depression or probable anxiety mediates the association between multimorbidity and ACE score. The results show that the ACE score positively predicts multimorbidity (total effect: β, 0.3417 [95% CI 0.3114; 0.3720]). Analysing the indirect effects, results reveal that probable depression (β, 0.0068 [95% CI 0.0008; 0.0128]) and probable anxiety (β, 0.0117 [95% CI 0.0048; 0.0188]) partially mediates the relationship between multimorbidity and ACE score. Nevertheless, the results suggest that even after accounting for the mediating effects of probable depression or probable anxiety, the ACE score still has a positive impact on multimorbidity. Additionally, probable depression or probably anxiety accounted for 2.0 and 3.4% of the total effect, respectively.

## Discussion

Data from this national SA survey confirmed a widespread prevalence of mental health risk among adults 18 years and older. Approximately one in every four South African respondents reported moderate to severe symptoms of probable depression. Seventeen percent of the population were anxious and 14.8% reported high exposure ACEs. This national survey also found that 21.6% of the population are living with two or more morbidities with the Eastern Cape Province reporting the highest prevalence of multimorbidity out of all nine provinces. Furthermore, a higher ACE score and several socio-demographics were associated with a higher likelihood of presenting with an adverse health profile of two or more morbidities and lastly, being depressed and/or being anxious was found to mediate the association between early adversity and multimorbidity in adulthood.

### Repeated cross-sectional findings

Using the PHQ-9 and GAD-7 surveys as indicators of probable depression and anxiety, respectively, we found that the percentage of respondents in Panel 2 who are probably depressed or anxious was comparable to those respondents in Panel 1 (probable depression: 26.2 vs. 25.7% and probable anxiety: 17.8 vs. 17.0%). The pandemic became a major source of mental illness resulting in the overall prevalence of mental ill health increasing worldwide (21). Our results are therefore indicative that the prevalence of mental health among South African adults during (Panel 1–2021) to post (Panel 2–2022) pandemic has not yet improved. The aftermath of the pandemic seems to be obscure. Recent studies support the existence of a mental health epidemic curve, describing the high probability increase in the burden of mental health during and post-pandemic (22–24), presumably exacerbated due to the resultant economic hardships (25).

The nexus of increasing prevalence of mental illness in low-resource settings and the increase in extreme poverty in LMICs in the context of the pandemic is apparent. Several studies have actively reported how measures such as isolation, social distancing and public restrictions severely impacted the mental health and general life of many South Africans (26–33). As shown in the results of Panel 1, poor mental health, poverty, and SES remain inextricably linked (5). The results of this repeated national survey not only show that the prevalence of probable depression and anxiety among South African respondents remains alarmingly high post-pandemic, but also this study confirmed that the prevalence of mental health is higher across several socio-demographics. For example, probable depression was higher among those widowed, divorced or separated, those with fewer resources and/or only completing a basic level of education such as primary school. Likewise, higher levels of probable anxiety, comparable to Panel 1, were reported in those with a marital status of widowed, divorced or separated, 65 years or older, and/or with primary school level education. Therefore, it seems that marital disruptions (34, 35), having less resources (i.e., fewer household amenities) and only having a basic level of education (36, 37) are not only linked to heightened levels of depression and/or anxiety in South African adults, but also are the sole demographics link to poverty within this setting.

Probable depression in Panel 2 was, however, found higher in younger respondents (aged 35-44 years) and not in those 65 years and older as previously reported in Panel 1 (5). The World Health Organisation estimated that one in five younger individuals struggle with mental health and warned that the more risk factors youth are exposed to, the greater the potential impact on their mental health (38). In a time where the vulnerable are exposed to heighted levels of bullying, social ostracization, family dysfunction, problems at school and/or in the workplace and trauma, may trigger a mental health issue. Probable depression was also now higher in those unemployed and who reside in the city and/or town areas. Comparable to a study conducted in the United States (US), younger adults (aged 18–39 years) experienced greater increases in high levels of depression and anxiety as a result of the pandemic (39–43). While our repeated national survey does not have a pre-COVID-19 baseline, we can, however, gather some indication of the effects of the pandemic on mental health and the subsequent economic turmoil. Some of the more noticeable effects of the pandemic include its succeeding financial pressure (i.e., unemployment and the increasing cost of living). According to the International Labour Organisation’s Youth Employment in Times of COVID report, globally, the youth have not escaped the economic and social impacts of the pandemic (44). With business closures as a result of economic crises in sectors that conventionally employ the youth labour force, younger individuals are often the first to be made redundant, shifted onto insecure work contracts or not employed at all (44). While SA has had a high unemployment rate for more than 4 decades (45, 46), the pandemic resulted in the loss of more than 2 million jobs in just 2020 alone (47). More recent reports highlighted that SA’s economy shed more than 500 million jobs in the third quarter of 2021, aggravating the already high unemployment trends for young adults in the country (48). Therefore, it is plausible that pre-existing inequalities within the country may have deepened due to the subsequent increase in household poverty and food insecurity, adding to mental health stressors. In general, poverty increases the risk of mental illness that consequentially increases the likelihood of an economic recession at an individual and family level (49).

In a US study, 73.1% of high school learners (younger than 18 years) reported at least one ACE during the pandemic (50). Social isolation, job loss, school closures and public restrictions unleashed during the pandemic may have exacerbated ACEs. Nearly 15% of the South African adult population reported high exposure ACEs in 2022. Notably, the highest reported type of ACE in Panel 2 was household dysfunction. We acknowledge the notion that older adults may minimise and/or fail to recall ACEs when compared to younger adults or adolescents (51) and this may have resulted in more of our younger sample reporting higher numbers of ACEs in Panel 2 in comparison to those in Panel 1. Needless to say, the pandemic’s indirect social and economic impact on family stress, coupled with the pandemic and its’ response disproportionately affected low-income populations, which are already predisposed to increased risk of reporting ACEs (52). In this national survey, our finding of probable depression or probable anxiety likely increased by 15.0 or 12.0% respectively, with each standard deviation increase in ACE score confirms and reiterates that mental wellbeing in childhood is pivotal in determining mental health outcomes in adulthood (53–56).

### Additional cross-sectional findings

In addition to the repeated cross-sectional analyses, this national survey also facilitated the investigation of multimorbidity. We report a widespread prevalence of multimorbidity across the nine South African Provinces, with the Eastern Cape Province reporting the highest number of morbidities. Morbidities of three or more were more frequently reported among females, those 65 years and older, being widowed, divorced or separated, those who were retired, living in the city or town area, with fewer resources and/or with only a basic level of education.

The Eastern Cape province of SA is the most deprived area in the country with a disproportionate burden of unemployment, poverty and disease (57). The finding of higher rates of adverse health in this province is in accordance with the previous findings of a spatial analysis of the prevalence of multimorbidity in SA, which found higher prevalence rates in the Eastern Cape (58). The latter study also revealed socioeconomic disadvantage reflected the spatial pattern of multimorbidity particularly in the Eastern Cape Province (58). The incidence of infectious and chronic diseases and malnutrition is also higher in this province than the national average and the distribution of immunisations and healthcare services is the lowest (59). Therefore, the higher socioeconomic disadvantage in this province, coupled with this province consisting of predominantly rural former homelands characterised by extremely high levels of poverty, deprivation, famine and an underdeveloped healthcare system, presumably are the potentiating drivers of the high multimorbidity prevalence we report in this province.

Age is accepted to be an important predictor of multimorbidity (60). Our findings of higher multimorbidity in older adults are in line with numerous studies conducted in adults 40 years and older in both HICs [Australia (61), Canada (62), China (63) and several countries across Europe (64)] and LMICs [Vietnam (65), Bangladesh (66) and Tanzania (67)]. Older adults are at higher health risk due to the presence of multiple chronic diseases (61, 63, 64, 68, 69). Other comparable results are the findings from a study performed in a LMIC setting which showed higher multimorbidity among females who are widowed, divorced or separated (70). The effects of marriage on single health conditions have been thoroughly examined, showing that those who are married in comparison to their single, divorced or widowed counterparts have decreased risk of developing metabolic syndrome (71), reduced morbidity and mortality associated with ischemic heart disease (72) and an overall better cognitive function (73). The higher prevalence of multimorbidity among women is also often correlated to survival bias which observes men having a shorter life expectancy than women (74). Furthermore, our findings are therefore in accordance with numerous studies such as (i) a population-based study in Brazil that found an increased risk of multimorbidity among illiterate populations (75); (ii) another that reported an inverse association between multimorbidity and the level of education (70); and (iii) a systematic review, with 24 cross-sectional studies that identified that individuals with less education had 64% more chance of reporting multimorbidity (76). In SA, inequalities in health, education and SES have been extensively reported with the poor being disproportionately affected (77, 78).

The trajectory from ACEs to adverse health outcomes in adulthood has received increasing attention in the past 2 decades. Numerous studies have since highlighted the links between ACEs to future health problems with reference to allostatic overload (79), that is, the cumulative burden of chronic stress and life events. This study found a significant association between ACE scores with multimorbidity outcomes across various models, even after adjusting for socio-demographic factors. Based on prior literature, this expected finding of those respondents who reported having two or more morbidities presenting with increased odds of ACE exposure is comparable to several population-based studies (80, 81).

Our results have established significant associations between probable depression, probable anxiety, and ACEs and similarly between ACEs and multimorbidity. We also showed that the relationship between ACEs and multimorbidity (chronic conditions excluding depression) was partly mediated by probable depression and/or probable anxiety. Prior studies found that older adults with multimorbidity are associated with mental health (82). Numerous theories and models [i.e., Beck’s Developmental Model of Depression (83) and the Psychological and Biological Pathways Model], suggest that depressive symptoms manifest from an individual’s negative interpretation of their experience or diseases can reduce one’s ability to cope with life events.

This repeated cross-sectional study must be interpreted in the context of its strengths and limitations. This study is strengthened by its use of nationally representative data, including respondents from all nine provinces of SA, weighted accordingly to be representative of SA’s adult population. Data collection was strengthened by the fact that the field staff went through extensive training. COVID-related information was not included as part of the survey items, thus as COVID-19 is known to impact various aspects of health, we acknowledge this a potential study limitation. Additionally, this national survey includes the use of self-reported questionnaires (both mental health and multimorbidity) which may pose recall bias (84). It is known that respondents will report experiences that are more socially acceptable and/or preferred thus, we acknowledge this as a limitation of this study. By including a measure of ACEs and multimorbidity, the study provides evidence for the contribution of early childhood adversity on multimorbidity, over and above contemporary socio-demographics.

In conclusion, this study confirmed a widespread prevalence of mental health across South African adults aged 18 years and older. We also confirm that mental health remains alarmingly high post-pandemic where more than a quarter of respondents are probably depressed, nearly one in every five respondents are anxious and 14.8% reported high exposure ACEs. This study also assessed multimorbidity among the South African population and found that 13.9% of respondents reported living with three or more morbidities and, those with two or more morbidities were found to have increased odds of early adversity, irrespective of differing socio-demographics. Early adversity was also related to multimorbidity partly via mental health. Areas of public health services and/or behavioural interventions to prevent and reduce the occurrence of ACEs in childhood may, in part, assist in lowering the risk of adverse health outcomes and mental ill-health in later life.

## Data availability statement

The original contributions presented in the study are included in the article/**Supplementary material**, further inquiries can be directed to the corresponding author.

## Ethics statement

The studies involving humans were approved by Human Research Ethics Committee (Non-Medical) of the University of the Witwatersrand, South Africa (H21/06/36). The studies were conducted in accordance with the local legislation and institutional requirements. The participants provided their written informed consent to participate in this study.

## Author contributions

AC, WM, AM, SD, and SN were involved in the conception and planning of the study and interpretation of the results. SN was responsible for oversight of data collection. AC carried out the data analyses and generated tables, interpreted the data, did the literature search, and the writing of the paper. All authors interpreted the data and made a significant contribution to the interpretation of the results. All authors were responsible for revising the manuscript and approving the submitted version.
