# Supplementary material for: Exploring the national prevalence of mental health risk, multimorbidity and the associations thereof: a repeated cross-sectional panel study
Source: Front Public Health. 2023 Oct 18;11:1217699. doi: 10.3389/fpubh.2023.1217699 (PMC10619674; doi:10.3389/fpubh.2023.1217699)
Supplement: Supplementary file 1 [file Table_1.docx]

**Supplementary Table S1. COVID-19 lockdown restrictions implemented across South Africa during the data collection of Panel 1 (2021) and Panel 2 (2022).**

|  | **Panel 1 (2021)** | **Panel 2 (2022)** |
| --- | --- | --- |
| Lockdown level | Adjusted alert level 2 | Alert level 1 |
| Date of implementation | 13^th^ September 2021 | 5^th^ April 2022 |
| Major restrictions | - Mandatory curfew in the place of residence from 23h00 to 04h00 daily. - Closing of establishments, whether indoor or outdoor, at 22h00 daily. - Social distancing of 1.5m at all gatherings. - Mandatory protocols for public areas (i.e., wearing of face masks that cover nose and mouth, hand washing and hand sanitisation). - Attendance of funerals and cremations limited to 50 people or less, or if the venue is unable to hold the required number of people, only 50% venue occupancy. - Night clubs closed to public. - 20 land borders reopened, 33 land borders remain closed. - International air travel is restricted to certain airports across the country (for fully vaccinated persons, or unvaccinated persons who provided a negative COVID test obtained not more than 72hours before date of travel). - Rail, ocean, air, and land transport permitted. - Public transport: short distance (<200km – 100% vehicle occupancy); long distance (>200km – 70% vehicle occupancy). - Sale of liquor is only permitted Monday to Friday 10h00 to 18h00, excluding weekends and public holidays. On site consumption of alcohol until 22h00 daily. | - mandatory curfew lifted. There will therefore be no restrictions on the hours of movement of people. - Mandatory protocols for public areas (i.e., wearing of face masks that cover nose and mouth, hand washing and hand sanitisation). - Attendance of funerals and cremations limited to 200 people or less, or if the venue is unable to hold the required number of people, only 50% venue occupancy. - Night clubs closed to public. - Any person who is a laboratory confirmed positive COVID-19 case and is symptomatic, must immediately seek treatment. - 21 land borders reopened, 32 land borders remain closed. - International air travel is restricted to certain airports across the country (for fully vaccinated persons, or unvaccinated persons who provided a negative COVID test obtained not more than 72hours before date of travel). - Rail, ocean, air, and land transport permitted. - Public transport: short distance (<200km – 100% vehicle occupancy); long distance (>200km – 70% vehicle occupancy). - Sale of liquor is only permitted until 23h00 daily. - General: A person who has a laboratory confirmed positive COVID-19 case and who is symptomatic must self-isolate; or be admitted to a health establishment for isolation, if he or she cannot self-isolate, for a period of 7 days in accordance with the requirements of self-isolation, or isolation, unless a longer period is recommended by a medical practitioner in order to prevent transmission. |

South African Government (adapted from www.gov.za/covid-19/about) .

**Supplementary Table S2. Mental health risk and multimorbidity of the South African adult population stratified by province.**

|  | | | **Provinces** | | | | | | | | |
| --- | --- | --- | --- | --- | --- | --- | --- | --- | --- | --- | --- |
|  | | | **WC** | **EC** | **NC** | **FS** | **KZN** | **NW** | **GP** | **MP** | **LP** |
| **Depression (PHQ9)** | **Probable depression** | % | 20.9 | 29.8 | 24.8 | 35.1 | 19.7 | 37.1 | 23.7 | 38.3 | 25.7 |
|  | Minimal | % | 50.6 | 48.2 | 41.5 | 26.9 | 58.1 | 40.7 | 54.6 | 41.4 | 42.1 |
|  | Mild | % | 28.5 | 22.1 | 33.8 | 38.0 | 22.1 | 22.2 | 21.6 | 20.3 | 32.2 |
|  | Moderate | % | 14.2 | 16.4 | 13.5 | 22.0 | 10.7 | 26.3 | 15.4 | 23.2 | 21.1 |
|  | Moderately severe | % | 4.7 | 9.8 | 7.7 | 6.7 | 7.2 | 8.8 | 5.9 | 12.8 | 4.2 |
|  | Severe | % | 2.0 | 3.6 | 3.6 | 6.4 | 1.8 | 2.0 | 2.4 | 2.3 | 0.4 |
| **Anxiety (GAD7)** | **Probable anxiety** | **%** | 14.6 | 20.3 | 20.4 | 22.6 | 13.6 | 20.3 | 15.7 | 24.1 | 14.7 |
|  | Minimal | % | 58.5 | 54.7 | 54.3 | 33.8 | 61.6 | 46.4 | 60.6 | 49.7 | 50.8 |
|  | Mild | % | 26.8 | 25.0 | 25.2 | 43.6 | 24.9 | 33.3 | 23.8 | 26.3 | 34.5 |
|  | Moderate | % | 11.6 | 15.7 | 6.5 | 17.2 | 10.6 | 15.7 | 12.7 | 18.4 | 13.6 |
|  | Severe | % | 3.0 | 4.6 | 13.9 | 5.4 | 3.0 | 4.6 | 3.0 | 5.7 | 1.1 |
| **ACEs** | ACE score | Mean ± SD | 1.77 ± 2.48 | 0.752 ± 1.65 | 2.76 ± 2.48 | 2.16 ± 3.96 | 0.850 ± 1.78 | 1.65 ± 3.02 | 1.27 ± 2.20 | 1.32 ± 2.16 | 2.29 ± 2.67 |
|  | No exposure | % | 47.1 | 72.1 | 21.1 | 68.4 | 70.6 | 63.2 | 63.9 | 57.9 | 38.4 |
|  | Intermediate exposure | % | 33.6 | 21.0 | 45.3 | 11.1 | 21.7 | 19.5 | 21.5 | 29.4 | 37.2 |
|  | High exposure | % | 19.3 | 6.9 | 33.7 | 20.5 | 7.7 | 17.3 | 14.7 | 12.7 | 24.4 |
| **Multimorbidity (chronic conditions incl. mental health)** | Multimorbidity score | Mean ± SD | 0.643 ± 1.29 | 0.808 ± 1.28 | 0.651 ± 1.16 | 0.504 ± 1.48 | 0.314 ± 0.706 | 1.08 ± 2.51 | 0.608 ± 1.41 | 0.323 ± 1.50 | 0.591 ± 0.739 |
|  | 0-1 morbidity | % | 81.9 | 78.3 | 85.6 | 88.2 | 92.3 | 82.9 | 85.7 | 85.0 | 93.5 |
|  | 2 morbidities | % | 9.5 | 12.0 | 5.9 | 3.9 | 5.4 | 5.8 | 6.9 | 7.9 | 2.6 |
|  | 3+ morbidities | % | 8.7 | 9.6 | 8.5 | 7.8 | 2.3 | 11.4 | 7.4 | 7.1 | 3.8 |

Probable depression was categorised into 5 groups based on scoring in the range 0-4 (minimal), 5-6 (mild), 10-14 (moderate), 15-19 (moderately severe), and 20-27 (severe). Probable anxiety was categorised into four groups based on scoring in the range 0-4 (minimal), 5-9 (mild), 10-14 (moderate), and 15-21 (severe). The ACE score was categorised into 3 exposure groups based on scoring 0 (no exposure); 1-3 (intermediate exposure) and 4-12 (high exposure). Multimorbidity score was categorised into 3 groups based on those respondents who reported null or one ailment (0-1 morbidity); those with comorbidity (2 morbidities); and those with multimorbidity (3 morbidities). Abbreviations: %: percentage; ACEs: adverse childhood experiences; PHQ9: Patient Health Questionnaire; GAD7: Generalised Anxiety Disorder; WC: Western Province; EC: Eastern Cape; NC: Northern Cape; FS: Free State; KZN: Kwa-Zulu Natal; NW: North West Province; GP: Gauteng Province; MP: Mpumalanga; LP: Limpopo Province.

**Supplementary Table S3: Logistic regressions to determine the odds of having probable depression or probable anxiety in adulthood.**

|  |  |  | **Depression binary (PHQ-9)**  (*n*= 3458) | | | **Anxiety binary (GAD-7)**  (*n*= 3458) | | |
| --- | --- | --- | --- | --- | --- | --- | --- | --- |
|  |  |  | **OR** | **(95 % Cl)** | ***p* value** | **OR** | **(95 % Cl)** | ***p* value** |
| **Model 1** | ACE | Score | 1.15 | (1.148; 1.149) | **<0.001** | 1.12 | (1.116; 1.117) | **<0.001** |
| **Model 2** | ACE | Score | 1.15 | (1.146; 1.147) | **<0.001** | 1.12 | (1.114; 1.115) | **<0.001** |
|  | Age | Years | 1.02 | (1.016; 1.016) | **<0.001** | 1.01 | (1.007; 1.007) | **<0.001** |
|  | Sex | Male | (reference) | | | (reference) | | |
|  |  | Female | 1.01 | (1.004; 1.007) | **<0.001** | 1.04 | (1.033; 1.037) | **<0.001** |
|  | Race | Black | (reference) | | | (reference) | | |
|  |  | White | 1.01 | (1.004; 1.010) | **<0.001** | 1.09 | (1.086; 1.093) | **<0.001** |
|  |  | Indian/Asian | 1.04 | (1.034; 1.044) | **<0.001** | 0.306 | (0.303; 0.308) | **<0.001** |
|  |  | Coloured | 0.877 | (0.0875; 0.880) | **<0.001** | 0.744 | (0.741; 0.746) | **<0.001** |
|  | Education | Uneducated/Partial primary | (reference) | | | (reference) | | |
|  |  | Primary | 1.31 | (1.305; 1.321) | **<0.001** | 1.48 | (1.470; 1.490) | **<0.001** |
|  |  | Partial secondary | 0.773 | (0.770; 0.776) | **<0.001** | 1.02 | (1.013; 1.023) | **<0.001** |
|  |  | NSC/Short course | 0.911 | (0.907; 0.915) | **<0.001** | 1.02 | (1.014; 1.024) | **0.013** |
|  |  | Tertiary | 1.20 | (1.198; 1.210) | **<0.001** | 1.16 | (1.148; 1.161) | **<0.001** |
|  | SES | Score | 0.956 | (0.956; 0.957) | **<0.001** | 0.966 | (0.965; 0.966) | **<0.001** |
|  | Employment | Unemployed | (reference) | | | (reference) | | |
|  |  | Employed | 0.822 | (0.821; 0.824) | **<0.001** | 0.871 | (0.869; 0.872) | **<0.001** |
|  |  | Student | 1.01 | (1.010; 1.016) | **<0.001** | 0.820 | (0.817; 0.823) | **<0.001** |
|  |  | Retired | 0.734 | (0.731; 0.736) | **<0.001** | 1.24 | (1.236; 1.247) | **<0.001** |
|  | Marital status | Single | (reference) | | | (reference) | | |
|  |  | Married/Co-habit | 0.687 | (0.686; 0.689) | **<0.001** | 0.706 | (0.705; 0.708) | **<0.001** |
|  |  | Widowed/Divorced/Separated | 1.03 | (1.030; 1.037) | **<0.001** | 1.07 | (1.070; 1.077) | **<0.001** |
|  | Urbanicity | Metropolitan | (reference) | | | (reference) | | |
|  |  | City/Towns | 1.16 | (1.155; 0.160) | **<0.001** | 1.17 | (1.164; 1.169) | **<0.001** |
|  |  | Rural/Village | 0.939 | (0.938; 0.941) | **<0.001** | 0.777 | (0.775; 0.779) | **<0.001** |

Model 1: regression unadjusted. Model 2: regression adjusted for socio-demographics. Abbreviations: *n* – number of participants; OR: odds ratio; ACE: adverse childhood experiences; SES: socioeconomic status; PHQ-9: Patient Health Questionnaire; GAD-7: Generalized Anxiety Disorder. Bold values denote statistical significance (p<0.05).

**Supplementary Table S4: Chronic conditions of the South African population stratified by province.**

|  |  | **Provinces** | | | | | | | | |
| --- | --- | --- | --- | --- | --- | --- | --- | --- | --- | --- |
|  |  | **WC** | **EC** | **NC** | **FS** | **KZN** | **NW** | **GP** | **MP** | **LP** |
| **Chronic conditions** | | | | | | | | | | |
| Heart disease | % | 1.3 | 3.4 | 1.1 | 3.4 | 0.3 | 5.4 | 3.8 | 7.2 | 1.2 |
| Stroke | % | 1.8 | 3.3 | 1.1 | 3.1 | 1.1 | 5.3 | 1.6 | 0.8 | 0.4 |
| High cholesterol | % | 10.2 | 13.7 | 13.4 | 3.4 | 5.9 | 12.7 | 12.5 | 10.1 | 7.2 |
| Diabetes | % | 6.8 | 10.3 | 2.7 | 9.2 | 4.1 | 10.0 | 7.2 | 4.3 | 2.2 |
| Overweight/obesity | % | 3.3 | 3.8 | 5.2 | 1.2 | 1.0 | 5.4 | 3.2 | 5.5 | 2.9 |
| HIV/AIDS | % | 4.6 | 4.6 | 1.5 | 4.3 | 3.8 | 10.7 | 3.5 | 9.4 | 2.7 |
| Asthma/chronic COPD | % | 4.6 | 7.1 | 3.4 | 2.9 | 1.2 | 10.8 | 3.9 | 5.1 | 4.6 |
| Sore joints/muscle problems | % | 10.2 | 9.9 | 19.0 | 7.0 | 4.9 | 7.2 | 8.1 | 3.7 | 3.5 |
| Tuberculosis | % | 4.1 | 1.1 | 4.3 | 2.9 | 1.4 | 5.6 | 1.6 | 4.4 | 0.9 |
| Cancer | % | 0.9 | 1.4 | 1.1 | 1.8 | 0.3 | 6.7 | 1.6 | 1.8 | 0.0 |
| Mental health | % | 3.5 | 2.3 | 6.6 | 2.0 | 1.8 | 4.5 | 2.7 | 3.9 | 0.7 |
| Liver disease | % | 0.5 | 1.3 | 0.0 | 1.8 | 0.3 | 5.4 | 0.8 | 0.3 | 1.5 |
| Chronic kidney disease | % | 0.8 | 1.5 | 0.0 | 1.8 | 0.9 | 4.5 | 1.5 | 3.1 | 0.6 |
| Hypertension | % | 11.7 | 16.9 | 5.9 | 5.6 | 4.5 | 13.3 | 8.7 | 7.9 | 3.7 |

Abbreviations: %: percentage; WC: Western Province; EC: Eastern Cape; NC: Northern Cape; FS: Free State; KZN: Kwa-Zulu Natal; NW: North West Province; GP: Gauteng Province; MP: Mpumalanga; LP: Limpopo Province; HIV: human immunodeficiency virus; AIDS: acquired immunodeficiency syndrome; COPD: chronic obstructive pulmonary disease.
